# Body Composition and Eating Habits in Newly Diagnosed Graves’ Disease Patients Compared with Euthyroid Controls

**DOI:** 10.3390/nu17233750

**Published:** 2025-11-28

**Authors:** Laura Croce, Cristina Pallavicini, Vittorio Gabba, Marsida Teliti, Alessandro Cipolla, Benedetta Gallotti, Pietro Costa, Benedetta Cazzulani, Flavia Magri, Mario Rotondi

**Affiliations:** 1 Department of Internal Medicine and Therapeutics, University of Pavia, 27100 Pavia, Italy; laura.croce@unipv.it (L.C.); marsida.teliti@unipv.it (M.T.); benedetta.gallotti01@universitadipavia.it (B.G.); pietro.costa01@universitadipavia.it (P.C.); flavia.magri@unipv.it (F.M.); 2 Unit of Endocrinology and Metabolism, Laboratory for Endocrine Disruptors, Istituti Clinici Scientifici Maugeri IRCCS, 27100 Pavia, Italy; cristina.pallavicini@icsmaugeri.it (C.P.); alessandro.cipolla01@universitadipavia.it (A.C.); 3 Department of Clinical-Surgical, Diagnostic and Pediatrics, University of Pavia, 27100 Pavia, Italy; vittorio.gabba@icsmaugeri.it; 4 Neurorehabilitation and Spinal Unit, Istituti Clinici Scientifici Maugeri IRCCS, 27100 Pavia, Italy; benedetta.cazzulani@icsmaugeri.it

**Keywords:** Graves’ disease, body composition, weight loss, thyrotoxicosis, standardized phase angle, bioelectrical impedance analysis

## Abstract

**Objectives**: Graves’ disease (GD) is the most common cause of hyperthyroidism and is associated with marked changes in body weight and body composition. Although weight loss is frequently reported, the extent and clinical relevance of body composition alterations, as well as their relationship with thyroid function, remain unclear. This study aimed to evaluate body composition and eating habits in patients with newly diagnosed hyperthyroid GD according to pre-morbid weight variation, and to compare these findings with those of matched euthyroid controls. **Methods**: Forty-four consecutive GD patients were enrolled and stratified based on the presence or absence of pre-morbid weight loss. Anthropometric measurements, thyroid function tests, thyroid volume, dietary habits (PREDIMED score, macronutrient intake and total daily caloric intake) and body composition assessed by bioelectrical impedance analysis (BIA) were collected. Standardized phase angle (SPA) and body cell mass index (BCMI) were calculated as nutritional indices. Body composition parameters and dietary adherence were compared with those of 44 age-, sex- and BMI-matched euthyroid controls. **Results**: Most GD patients (70.3%) reported weight loss before diagnosis; however, the magnitude of weight change did not correlate with the biochemical severity of thyrotoxicosis. Patients without weight loss showed higher fat mass percentage and higher caloric intake than those who lost weight. SPA was significantly associated with FT3, FT4 and TRAb levels, independently of age, sex, BMI and fat mass. Compared with controls, GD patients exhibited lower phase angle and SPA, higher extracellular water percentage and reduced BCMI, whereas fat mass and adherence to the Mediterranean diet were similar. **Conclusions**: Hyperthyroid GD patients display increased extracellular water and reduced body cell mass. SPA is inversely associated with GD severity and represents a valuable clinical tool for assessing nutritional status in thyrotoxic patients. Pre-morbid weight changes are not proportional to disease severity and may instead reflect increased caloric intake.

## 1. Introduction

Graves’ disease is an autoimmune thyroid disorder and the most common cause of hyperthyroidism. Body weight changes are a major concern among individuals with thyroid disorders. While hypothyroidism is generally associated with weight gain, hyperthyroidism is more frequently linked to weight loss during the active phase of the disease.

Hyperthyroidism leads to increased thermogenesis, oxygen consumption and metabolism, resulting in weight loss in about 90% of younger patients, with an expected reduction of 5–7 kg compared to the weight one year prior to diagnosis [1,2,3]. Paradoxically, some patients are reported to gain weight during hyperthyroidism. This is likely due to increased appetite and caloric intake exceeding the metabolic rate. Because objective pre-morbid weight records are usually unavailable, weight variation is typically based on patient self-report.

Hyperthyroidism affects body composition, with the most widely reported alteration being a reduction in lean body mass [4,5,6]. Nevertheless, the interpretation of body composition data is limited by the high heterogeneity of study design, small samples sizes and the different instruments used for body composition analysis.

Bioelectrical impedance analysis (BIA) is a quick, cheap and widely available technique to assess body composition in healthy subjects and in the context of several diseases. It is a technique particularly suitable to assess derangements in body fluids and cell membrane integrity [7]. This technique was used in some studies evaluating the alterations in body composition in GD patients, showing a tendency towards an increase in extracellular water (ECW) and a reduction of body cell mass (BCM) in hyperthyroid GD patients when compared with controls [8,9]. A recent study by Sciacchitano et al. evaluated body composition in a small sample of patients with overt hyperthyroidism, confirming a tendency towards an excess in ECW [10]. Nevertheless, these studies included small samples of patients and did not evaluate eating habits and body weight variations before the onset of disease. Moreover, BIA data can be impacted by age and gender. Since GD can occur both in men and women and in a wide age range, novel body composition indexes that take into account these variables are needed.

The aim of the present study was to (i) evaluate the body composition and eating habits of a group of GD patients in the hyperthyroid phase of disease and their relationship with thyroid function parameters and pre-morbid body weight variations (ii) compare body composition parameters and adherence to the Mediterranean diet of hyperthyroid GD patients with an age-, gender- and BMI-matched control group of euthyroid controls.

## 2. Materials and Methods

### 2.1. Patients

For the aim of the present study, patients newly diagnosed with Graves’ disease attending the Outpatient Endocrinology and Metabolism Unit of ICS Maugeri I.R.C.C.S. (Pavia) were enrolled. Inclusion criteria were as follows: (i) diagnosis of Graves’ disease in an endocrinology setting based on the presence of thyrotoxicosis and positive TRAb antibodies, (ii) BMI > 16 kg/m^2^ and < 35 kg/m^2^, (iii) Age > 18 years, (iv) and agreeing to enroll in the study. Exclusion criteria were as follows: (i) Relapse of Graves’ disease or thyrotoxicosis not related to Graves’ disease; (ii) any serious cardiovascular or renal event in the previous 6 months; (iii) ongoing pregnancy or breastfeeding; (iv) any condition potentially leading to fluid overload such as heart failure (NYHA class > I) or liver cirrhosis; (v) a known cause of malabsorption (including uncontrolled celiac disease, lactose intolerance or inflammatory bowel disease); and (vi) ongoing therapy with weight-modifying medications (e.g., GLP-1 analogs, metformin).

A control group of subjects without a personal history of thyroid diseases was enrolled. For this group, the same inclusion and exclusion criteria (apart from those related to Graves’ disease) were employed. A 1:1 case–control matching was performed to balance the two groups (Graves’ disease vs. controls) based on age, sex and body mass index (BMI). Sample size was calculated based on an expected difference in PhA value between GD patients and controls of at least 0.5° with a standard deviation of 0.6°, a 90% power and a double-sided 95% confidence interval. The required sample size was of at least 32 patients per group.

The final study group included 44 patients with active GD (GD group) and 44 controls of similar age, gender and BMI.

All patients had signed an informed consent concerning the future use of their clinical-pathological data for research purposes. The present study was approved by the Ethical Committee “Comitato Etico Territoriale Lombardia 6” (Protocol N. 0065286/23).

### 2.2. Anthropometric Measurements

Weight was measured using two different calibrated scales, whose accuracy was periodically verified by a standardization body. Participants stood still in the center of the scale platform, wearing light clothing, with heels 10 cm apart and body weight evenly distributed on both legs. Height was measured with a stadiometer to the nearest 0.1 cm, with participants standing upright, barefoot and with heels on the floor against the instrument. Body mass index (BMI) was calculated as weight in kilograms divided by height in meters squared. Data regarding heart rate at diagnosis (reported as beats per minute, bpm) was also collected.

### 2.3. Thyroid Function Parameters

For patients’ in the Graves’ disease group, data regarding serum anti-TSH receptor antibodies (TRAb), free triiodothyronine (FT3) and free thyroxine (FT4) were collected. TRAb, FT3 and FT4 were measured using the Alinity I system (Abbott Laboratories, Chicago, IL, USA), an automated chemiluminescent microparticle immunoassay (CMIA). The method employs paramagnetic microparticles coated with anti-analyte antibodies and acridinium-labeled conjugates.

Measurement intervals were 0.42–5.00 ng/dL for FT4, 1.5–20 pg/mL for FT3, 1.25–50.00 U/L for TRAb. Intra-assay coefficients of variation (CVs) ranged from 1.7 to 3.0% for FT4, from 2.4 to 3.8% for FT3, from 1.1 to 4.8% for TRAb. Intra-laboratory CVs ranged from 2.0 to 3.1% for FT4, from 3.6 to 4.8% for FT3, from 1.2 to 5.2 for TRAb. Limit of Blank (LoB) was 0.22 ng/dL for FT4, 0.88 pg/mL for FT3, 0.38 U/L for TRAb. Limit of Detection (LoD) was 0.28 ng/dL for FT4, 0.95 pg/mL for FT3, 0.70 U/L for TRAb. Limit of Quantitation (LoQ) was 0.42 ng/dL for FT4, 1.25 pg/mL for FT3, 1.26 U/L for TRAB. Reference ranges were: FT3 1.71–3.71 pg/mL, FT4 0.70–1.48 ng/dL, TRAb < 1.5 U/L. Quality-control pools at low, normal and high concentrations were included in each assay.

### 2.4. Thyroid Volume

Thyroid ultrasound was performed using a real-time ultrasound device equipped with a 7.5 MHz linear transducer. All examinations were conducted by the same experienced operator. Thyroid volume was calculated using the ellipsoid formula: length × width × depth × 0.479.

### 2.5. Body Composition Analysis

Body composition was assessed in the fasting state using bioelectrical impedance analysis (BIA) with an Akern BIA 101 analyzer (Akern Srl, Pontassieve, Italy). Parameters obtained included phase angle (PhA), extracellular water percentage (ECW%), fat mass % (FM%), body cell mass (BCM), Muscle Mass percentage (%) and Resting Energy Expenditure (REE). Based on these parameters, two derived nutritional indexes were calculated.

- Body cell mass index (BCMI): obtained dividing BCM by the square height in meters (BCM/height^2^) [11].
- Standardized phase angle (SPA): calculated as [(observed PA—mean PA)/standard deviation of PA], providing Z scores for PA values [12]. This index provides an estimate of the deviation from the correspondent PhA in the healthy population, adjusted for age, sex and BMI [13,14].

Data were analyzed using Bodygram Plus software (Akern Srl, Pontassieve, Italy).

### 2.6. Dietary Assessment

All patients underwent an individual dietary-nutritional evaluation conducted by a registered clinical dietitian. Dietary intake and adherence to the Mediterranean diet were evaluated using the PREDIMED (Prevención con Dieta Mediterránea) questionnaire, administered by a trained nutritionist, in both GD patients and controls. Each question was scored 0 or 1, and the total PREDIMED score was calculated as the sum of all items. A score of 0–5 indicated low adherence, 6–9 moderate adherence and ≥10 high adherence to the Mediterranean diet. Moreover, a dietary history was collected in order to identify possible pathological eating behaviors. In GD patients, daily energy intake and dietary intake of protein, fat, carbohydrates and water was assessed through a 24 h dietary diary and classified as normal, excessive or scarce. In GD patients, the dietician collected information in detail from each patient, including the estimated weight variation between before symptom onset and the moment of evaluation.

### 2.7. Statistical Analysis

Statistical analysis was performed using the SPSS Software 30.0 (SPSS, Inc., Chicago, IL, USA). Between-groups comparisons were performed using the Student’s *t*-test for unpaired data and the Mann–Whitney U-test according to a normal or a non-parametric distribution. Within-group comparisons were performed using the Student’s *t*-test for paired data and the Wilcoxon’s test according to a normal or a non-parametric distribution. Frequencies among groups were performed using the χ^2^-test with Fisher’s correction when appropriate. Correlations among continuous variables were assessed using Spearman’s rank correlation coefficient (rho), given the non-normal distribution of several parameters. For each pair of variables, both the correlation coefficient (rho) and the corresponding *p*-value were computed. To evaluate the association between SPA and thyroid-related variables (FT3, FT4, TRAb) independently of potential confounders, a linear regression model was designed including SPA as a dependent variable and age, gender, BMI and FM% as dependent variables. Statistical significance was defined as *p* < 0.05.

## 3. Results

### 3.1. Clinical, Anthropometric and Biochemical Characteristics and Body Composition of the GD Group

The 44 patients (33 females and 11 males) with active GD had a mean age of 47.4 ± 15.2 years, a mean BMI of 23.1 ± 4.5. Median FT3 levels were 6.9 (4.5–14.3) ng/dL, median FT4 levels were 20.7 (17.0–30.5) ng/dL and TRAb levels were 9.8 (4.4–16.9) U/L. Median thyroid volume was 13 mL (12–18). Ten patients were taking a non-thyroid related medication, including beta-blockers (six patients), calcium antagonists (four patients), statins (two patients) and progestinic birth-control pills (two patients). No patients were taking body weight modifying therapies. When asked about the body weight variations that occurred before disease onset, patients reported a median body weight reduction of 2.8 (0.1–6.5) kg, corresponding to a percentage change when compared to pre-morbid weight of 4.2 (0.2–7.8)%.

Evaluation of eating habits revealed that four patients (9.5%) had restrictive eating behaviors.

Patients were stratified according to the reported weight variation before disease onset. As shown in Table 1, 32 patients (72.7%) reported weight loss, with a median reduction of 6.5 (3.7–10.6) kg, corresponding to a percentage reduction of 4.0 (2.5–7.4)%. Twelve patients (27.3%) reported a stable or increased body weight, with a median increase of 0.5 (0–2.1) kg, corresponding to a percentage increase of 0.4 (0.0–3.4)%.

As shown in Table 1, the two groups were similar in terms of age, gender, BMI at the moment of diagnosis, BMI before disease onset, PhA, SPA, ECW%, BCMI and percentage of patients who performed regular physical activity. GD patients who did not lose weight had a higher fat mass percentage and lower Muscle Mass percentage. While the Resting Energy Expenditure was similar between the two groups, estimated daily caloric intake was significantly higher among patients who did not lose weight. No differences in terms of macronutrients intake or adherence to the Mediterranean diet (as assessed through the PREDIMED questionnaire) was observed. FT3 and FT4 levels were similar in the two groups, while a higher TRAb titer was observed in patients who did not lose weight.

### 3.2. Correlation Between Severity of Hyperthyroidism and Clinical, Anthropometric and Biochemical Characteristics, Body Composition and Eating Behavior in the GD Group

**Figure 1:**
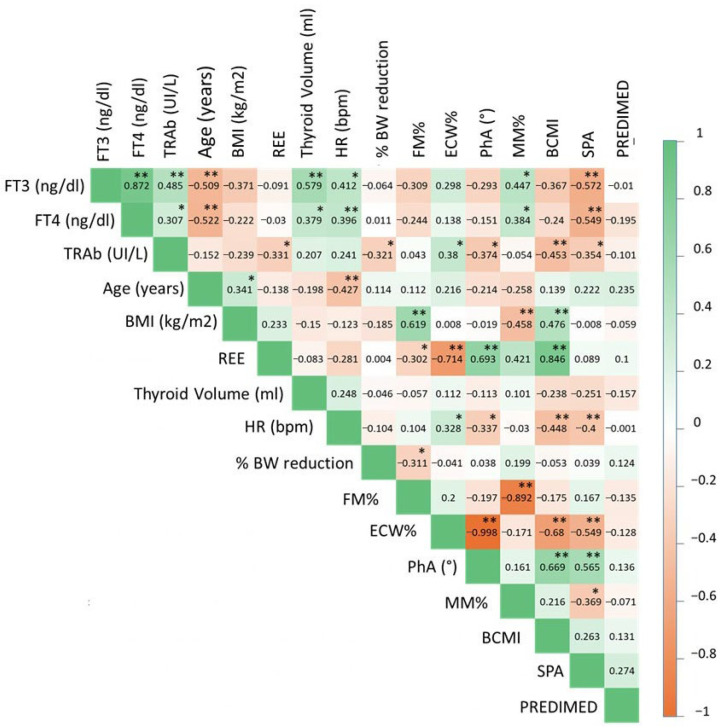
Heatmap of the pairwise Spearman’s correlation coefficients (rho) between continuous variables. The strength of correlation is represented by the color scale (red = negative correlation, green = positive correlation). Only the upper triangular portion of the correlation matrix is displayed to avoid redundancy. * *p* < 0.05; ** *p* < 0.001).

As shown in Figure 1, a Spearman correlation between FT3, FT4 and TRAb levels and age, BMI, percentage of pre-morbid weight loss, body composition parameters, REE, heart rate, thyroid volume and PREDIMED score was performed. A significant bivariate non-parametric correlation could be observed between FT3 levels and age, heart rate, %MM and SPA values, between FT4 and age, thyroid volume, heart rate, MM% and SPA and between TRAb levels and REE, percentage of body weight reduction, PhA, SPA, BCMI and ECW%. No significant correlation between PREDIMED scores and reported percentage of body weight changes before the onset of disease with thyroid function parameters or body composition could be observed. SPA was the parameter that was most strongly and consistently correlated with thyroid function parameters and TRAb.

To confirm these findings, a linear regression analysis was performed including SPA as a dependent variable and thyroid-related parameters, age, gender, BMI and FM%, as covariates. As shown in Table 2, a significant linear inverse correlation between SPA and FT3, FT4 and TRAb could be observed independently from age, gender, BMI and FM%.

### 3.3. Comparison of Body Composition and Adherence to the Mediterranean Diet Between GD Patients and Controls

As shown in Table 3, GD patients and controls did not significantly differ in terms of age, gender and BMI. PREDIMED score showed similar adherence to Mediterranean diet between GD patients and controls. The comparison of body composition analysis showed that GD patients had significantly lower phase angle values, higher ECW% and lower BCMI. FM%, and conversely fee fat mass (FFM%), and REE were similar between the two groups.

**Figure 2:**
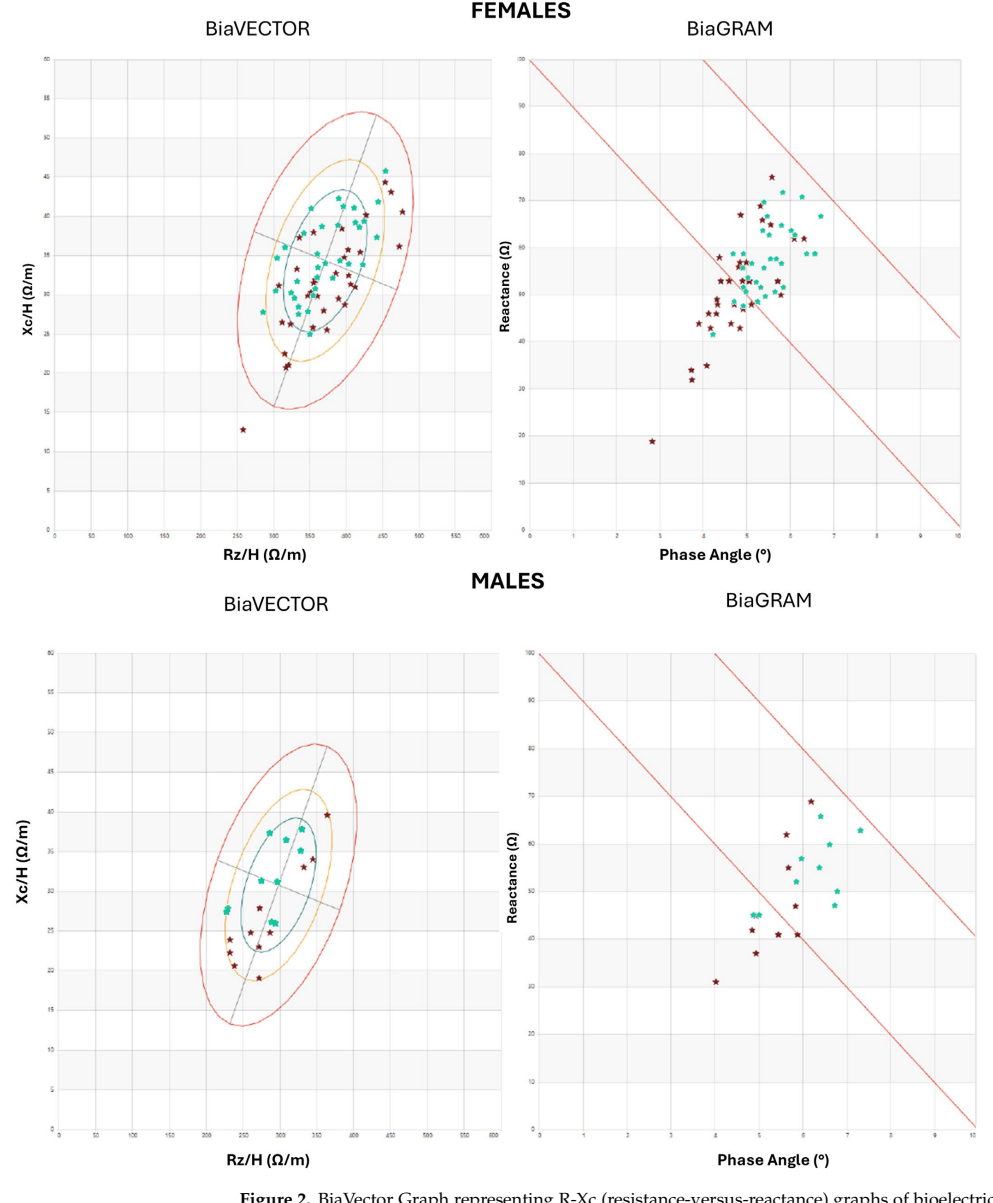
BiaVector Graph representing R-Xc (resistance-versus-reactance) graphs of bioelectrical impedance vector analysis and BiaGram Graphs representing the graphical relation between reactance and phase angle. Dark red stars represent GD patients, while green stars represent controls. The green oval represents 50th centile, the yellow oval the 75th centile, the red oval the 95th centile.

Differences in body composition between the two groups are shown in the BiaGram and BiaVector Graphs (Figure 2).

## 4. Discussion

The results of the present study show that while most patients with GD lose weight before disease onset, a relevant percentage of patients do not report any weight loss. Weight variations are not significantly related to the severity of thyrotoxicosis, TRAb levels, age or BMI. Patients who do not lose weight have a higher FM% compared to those who lost weight, with similar REE and body water distribution. While the two groups were similar in terms of adherence to the Mediterranean diet and macronutrient consumption, a slightly higher daily caloric intake was reported by patients who did not lose weight.

We demonstrated a significant correlation between the severity of thyrotoxicosis and TRAb titer and alterations in body composition, particularly an increase in extracellular water and reduction in body cell mass. Among the assessed parameters, SPA emerged as the body composition index that was most significantly and consistently related with both FT3, FT4 and TRAb levels. The significant inverse correlation was observed even after correction for confounders such as age, gender, BMI and FM%.

A comparison of GD patients with an equal number of gender-, age- and BMI-matched euthyroid controls showed that GD patients had a higher percentage of extracellular water and a lower body cell mass, with similar adherence to the Mediterranean diet.

These body composition alterations resemble those observed in catabolic or inflammatory states [15]. These findings are in part in line with the previous literature assessing this topic. A 1997 study by Seppel et al. evaluated 47 hyperthyroid GD patients, showing an increase in the extracellular mass/body cell mass (ECM/BCM) ratio compared with euthyroid controls, with no difference in lean body mass. Moreover, an inverse correlation between FT3 levels and phase angle could be observed [8]. On the other hand, a 1995 study by Hu et al. comparing 11 women with GD with 49 healthy controls found no significant differences in terms of BMI, percentage body fat (BF/BW), percentage lean body mass (LBM/BW) and percentage total body water (TBW/BW) between the two groups. GD patients had a lower body cell mass and a higher percentage of extracellular water. FT3 and FT4 levels were inversely related with BCM values and directly related with ECW% [9]. Similarly, a 1999 study by Miyakawa et al. observed that the ECM (extracellular mass)/BCM (body cell mass) ratio was significantly increased in GD patients when compared with controls, which was the result of marked depletion of BCM with concomitant expansion of ECM [16]. These early findings were in part confirmed by a recent study by Sciacchitano et al., showing a non-linear U-shaped correlation between thyroid hormone concentration and several Bioimpedance parameters indicating an excess in extracellular body water, including PhA, TBW/FFM ratio and Na/K ratio [10].

Compared with the previous literature, the present study provides some novel insights on this topic. First, the present data show that SPA is inversely related with the severity of GD (both in terms of thyrotoxicosis and of TRAb levels) but also differentiates GD patients from euthyroid controls, independently from age, gender and BMI.

This index has been used as a predictor of nutritional status in several clinical contexts, such as transplant patients [17], elderly patients [18], surgical patients [19], patients with COVID-19 [20] and oncologic patients [21]. The standardized nature of this parameter (which is a Z-score comparing each patient to a population of age and gender match controls) allows for more reproducible results and to compare patients with different age and genders. Although GD is more common among women, 20–30% of GD patients are men, and the disease can occur in a wide age range. For this reason, SPA is a particularly useful index to assess body composition derangements in the context of GD thyrotoxicosis. Indeed, SPA values were inversely proportional to FT3, FT4 and TRAb levels even among a group of patients all characterized by a certain derangement of ECW%. Moreover, our findings further challenge the notion that thyrotoxicosis would mainly impact lean mass, as previously believed [4,5,6], rather supporting its effects on body water distribution.

As a second novel finding, our results showed that while most patients reported weight loss before the diagnosis of GD, the degree of weight loss was not proportional to the severity of thyrotoxicosis nor to the degree of alteration in the body composition parameters typically altered by thyrotoxicosis. Paradoxically, slightly higher TRAb levels were observed in the patients who did not lose weight. The only observed differences between patients who did or did not lose weight were a slightly higher FM%, a lower %MM and a higher caloric intake, despite similar diet composition. This finding suggests that body weight variations are the results of complex modifications in metabolic expenditure and eating and exercise patterns during the thyrotoxic phase of the disease [22,23,24]. In particular, patients not losing weight might be the ones in which the increase in appetite exceeds the increase in metabolic expenditure. Indeed, our results showed for the first time that patients who do not lose weight have a higher mean caloric intake when compared with those who lose weight, even with similar diet composition. These patients may be at particular risk of weight rebound after the restoring of euthyroidism. It is relevant to note that even patients who reported small or absent body weight variations prior to diagnosis had sometimes severe derangements in body composition. Thus, the degree of weight loss alone should not be regarded as indicative, per se, of the detrimental impact of thyrotoxicosis on body composition.

As a last observation, dietitian counseling led to the diagnosis of restrictive eating behavior in 4 out of 44 GD patients (8%). The presence of a restrictive eating disorder can be challenging in patients with GD, since this condition can lead to scarce therapeutic compliance in order to avoid weight regain [25]. This finding highlights the usefulness of a dietitian counseling in GD, since endocrinologist may not have the technical skills to detect eating behavior disorders in their patients.

This study has some limitations. The relatively small sample size and monocentric nature of the study may limit the power of our results. Moreover, BIA can be sensitive to small variations in hydration status, fasting compliance, electrode placement and instrumentation. The lack of follow-up data after restoring of euthyroid does not allow us to draw conclusions on the prognostic significance of our findings. Lastly, a complete dietary assessment with macronutrient composition and daily caloric intake was available only for GD patients and not controls, so a complete comparison of dietary habits between the two groups was not feasible.

Nevertheless, the present results support, under a clinical point of view, the usefulness of a nutritional assessment with body composition analysis in patients with GD. This evaluation allows the identification of patients with profound body weight composition alterations who might need nutritional support. In this context, SPA can be considered as the most reliable body composition index in GD patients. Moreover, the early identification of patients with restrictive eating disorders allows clinicians to manage these patients with particular attention.

## 5. Conclusions

The results of the present study demonstrate that patients with newly diagnosed hyperthyroid Graves’ disease exhibit profound alterations in body composition, characterized by increased extracellular water and reduced body cell mass, resembling patterns seen in malnutrition and systemic inflammation. Importantly, the standardized phase angle emerged as the most sensitive and consistent parameter, showing a strong inverse association with thyroid hormone levels and TRAb titers, independently of age and gender. These findings indicate that body weight variations alone are insufficient to capture the metabolic and nutritional burden of thyrotoxicosis. Instead, the assessment of phase angle and standardized phase angle by bioelectrical impedance analysis may provide a valuable, non-invasive tool for evaluating nutritional and functional status in this patient population. Future prospective studies are warranted to explore the mechanisms underlying these alterations, assess their reversibility with treatment, evaluate the prognostic value of these indices and determine their potential role in guiding individualized management strategies for Graves’ disease.

## Figures and Tables

**Table 1:** Comparison of clinical characteristics, body composition, eating habits, physical activity and thyroid function between GD patients who lost weight before disease onset and those who did not lose weight. BCMI: body cell mass index; BMI: body mass index; ECW: extracellular water; FM: fat mass; GD: Graves’ disease; MM: Muscle Mass; PhA: phase angle; SPA: standardized phase angle; FT3: free triiodothyronine; FT4: free thyroxine, TRAb: TSH receptor antibodies; bpm: beats per minute.

	GD Patients Who Lost Weight (32)	GD Patients Who Did Not Lose Weight (12)	*p* Value
Age (Years, mean ± SD)	47.0 ± 15.4	48.4 ± 15.4	0.792
Gender (Male/Female, % of Males)	10/22 (31.3%)	1/11 (8.3%)	0.118
BMI at diagnosis (kg/m^2^, mean ± SD)	22.6 ± 4.1	24.4 ± 5.3	0.234
Pre-morbid BMI (kg/m^2^, mean ± SD)	24.7 ± 4.7	24.0 ± 4.9	0.645
Body weight variation (kg, median, 25–75° centile)	−6.5 (−3.7–10.6)	0.5 (0.0–2.1)	**<0.001**
% Body weight variation (%, median, 25–75° centile)	−4.0 (−2.5–−7.4)	0.4 (0.0–3.4)	**<0.001**
Heart rate (bpm, mean ± SD)	86.3 ± 15.6	91.9 ± 20.9	0.348
PhA (°, mean ± SD)	5.0 ± 0.7	4.8 ± 0.8	0.604
SPA (median, 25–75° centile)	−0.4 (−1.5–0.4)	−0.3 (−1.3–0.4)	0.687
ECW (%, mean ± SD)	51.4 ± 4.5	52.2 ± 4.6	0.590
BCMI (mean ± SD)	8.2 ± 1.5	7.8 ± 1.1	0.407
FM% (%, mean ± SD)	23.1 ± 9.4	30.5 ± 9.0	**0.024**
MM% (%, mean ± SD)	37.5 ± 8.0	31.7 ± 5.9	**0.027**
Resting Energy Expenditure (kcal/day, mean ± SD)	1412.1 ± 160.5	1340.7 ± 87.5	0.153
Regular physical activity (N, %)			
Estimated daily caloric intake (kcal/day, mean ± SD)	2053.8 ± 252.9	2331.6 ± 183.4	**<0.001**
PREDIMED score (median, 25–75° centile)	6 (6–10)	6 (5–8)	0.490
PREDIMED adherence level (N, %)			0.471
Low	7 (21.9%)	3 (25.0%)	
Moderate	17 (53.10%)	8 (66.7%)	
High	8 (25.0%)	1 (8.3%)	
Fat intake (reduced, adequate, increased, N %)	3 (9.4%), 14 (43.8%), 15 (46.9%)	0 (0.0%), 9 (75.0%), 3 (25.0%)	0.151
Carbohydrate intake (reduced, adequate, increased, N %)	5 (15.6%), 19 (59.4%), 8 (25.0%)	2 (6.7%), 8 (66.7%), 2 (16.7%)	0.840
Fiber intake (reduced, adequate, N %)	21 (77.8%), 11 (34.4%)	6 (50.0%), 6 (50.0%)	0.343
Water intake (reduced, adequate, increased, N %)	9 (28.1%), 23 (71.9%)	3 (25.0%), 9 (75.0%)	0.836
FT3 (pg/mL, median, 25–75° centile)	6.8 (4.5–11.2)	9.0 (4.3–17.7)	0.727
FT4 (pg/mL, median, 25–75° centile)	20.8 (16.9–31.1)	21.0 (18.1–30.5)	0.969
TRAb (U/L, median, 25–75° centile)	7.5 (3.3–12.9)	16.0 (11.1–17.1)	**0.011**

Statistically significant values (*p* < 0.05) are shown in bold.

**Table 2:** Results of linear regression analysis model with SPA (standardized phase angle) as dependent variable and age, gender, BMI, FM% and thyroid function parameters (FT3, FT4, TRAb). The table shows the results of the linear regression for FT3, FT4 and TRAb after adjustment for age, gender, BMI and FM%.

	β	C.I. for β		*p* Value
		Lower	Upper	
FT3	−0.446	−0.861	−0.031	**0.036**
FT4	−0.445	−0.778	−0.111	**0.010**
TRAb	−0.332	−0.619	−0.045	**0.025**

Statistically significant values (*p* < 0.05) are shown in bold.

**Table 3:** Comparison of clinical characteristics, body composition and eating habits between GD patients and controls. BCMI: body cell mass index; BMI: body mass index; ECW: extracellular water; FM: fat mass; GD: Graves’ disease; MM: Muscle Mass; PhA: phase angle; SPA: standardized phase angle.

	GD Patients (44)	Controls (44)	*p* Value
Age (years, mean ± SD)	47.4 ± 15.2	43.4 ± 16.7	0.240
Gender (Male/Female, % of Males)	11/33 (25.0%)	10/34 (22.7%)	0.803
BMI at diagnosis (kg/m^2^, mean ± SD)	23.1 ± 4.5	22.9 ± 3.3	0.816
Resting Energy Expenditure (kcal, mean ± SD)	1392.6 ± 146.8	1456.6 ± 154.8	0.050
PhA (°, mean ± SD)	4.9 ± 0.7	5.6 ± 0.7	**<0.001**
SPA (median, 25–75° centile)	−0.4 (−1.3–0.4)	0.6 (−0.1–1.0)	**<0.001**
ECW (%,mean ± SD)	51.6 ± 4.5	47.6 ± 3.5	**<0.001**
BCMI (mean ± SD	8.1 ± 1.4	9.0 ± 1.5	**0.004**
FM% (mean ± SD)	25.1 ± 9.8	23.8 ± 7.0	0.461
MM% (mean ± SD)	35.9 ± 7.8	36.4 ± 6.3	0.780
PREDIMED score	6 (6–9)	7 (6–9)	0.251
PREDIMED adherence level (N, %)			0.451
Low	10 (22.7%)	8 (18.2%)	
Moderate	25 (56.8%)	30 (68.2%)	
High	9 (20.5%)	6 (13.6%)	

Statistically significant values (*p* < 0.05) are shown in bold.

## Data Availability

The data presented in this study are available on request from the corresponding author due to privacy issues.

## Abbreviations

The following abbreviations are used in this manuscript:

BCM, Body Cell Mass; BCMI, Body Cell Mass Index; BIA, Bioelectrical Impedance Analysis; BMI, Body Mass Index; bpm, beats per minute; COVID-19, Coronavirus Disease 2019; ECM, Extracellular Mass; ECW, Extracellular Water; FFM, Fat-Free Mass; FM, Fat Mass; FT3, Free Triiodothyronine; FT4, Free Thyroxine; GD, Graves’ Disease; LoB, Limit of Blank; LoD, Limit of Detection; LoQ, Limit of Quantitation; MM, Muscle Mass; NYHA, New York Heart Association; PhA, Phase Angle; PREDIMED, Prevención con Dieta Mediterránea; REE, Resting Energy Expenditure; SPA, Standardized Phase Angle; TBW, Total Body Water; TRAb, TSH receptor antibodies.
